# New Perspectives on Tooth Wear

**DOI:** 10.1155/2012/287573

**Published:** 2012-03-28

**Authors:** Peter W. Lucas, Ridwaan Omar

**Affiliations:** ^1^Department of Bioclinical Sciences, Faculty of Dentistry, Kuwait University, P.O. Box 24923, Safat 11310, Kuwait; ^2^Department of Restorative Sciences, Faculty of Dentistry, Kuwait University, P.O. Box 24923, Safat 11310, Kuwait

## Abstract

Some of the efforts that have been made to document tooth wear are reviewed here with an emphasis on nonhuman mammals, literature with which dentists may not be very familiar. We project a change in research strategy from the description of wear at various scales of measurement towards investigation of the mechanical mechanisms that actually create the texture of a worn surface. These studies should reveal exactly how tooth tissue is lost and what aspects of the structure of dental tissues affect this. The most important aspects of the interaction between the tooth surface and wear particles would appear to be particle size, particle shape, their mechanical properties with respect to those of tooth tissues, and the influence of saliva.

## 1. Introduction

The teeth of humans are used several thousand times a day [1], a figure that is probably an order of magnitude lower than in our nearest primate relatives [2]. This newly discovered discrepancy has been attributed to the effects of cooking food [3], which reduces the importance of food breakdown in the mouth [4]. Even so, humans make millions of potentially damaging mechanical contacts in a lifetime, and there are no current solutions to the engineering of any material that could compare to the exceptional damage tolerance of human dental enamel [5, 6].

Damage to teeth via contact with either ingested foods, extraneous particles ingested with them, or opposing teeth takes place at various scales. The largest events can lead to catastrophic fractures of the tooth crown or roots, fragmenting them [7]. The mechanisms that produce at least some types of crown fracture are now beginning to be elucidated and show that preexisting cracks in enamel are often implicated [8, 9]. An exception is seen in millimetre-scale enamel chipping, where it is clear that no preexisting flaws are involved [10]. However, a much more common result than these events is crown wear, which is the loss of volume caused by the accumulated loss of microscopic amounts of hard tissue over an extended time period. Such tooth wear is an enormously important aspect of oral biology, of interest to many researchers on vertebrates. However, this range of research interest has resulted in knowledge being spread across a wide range of journals. Little of this comparative interest is apparent in the dental literature. While, for the most part, wear studies in nonhumans present wear as a mechanical process [11, 12], the accepted view in dentistry seems to be that chemical dissolution is always involved [13–16]. Despite this discrepancy, the final removal of tooth tissue even in modern humans nearly always involves a force, leading to the need for a mechanical analysis, albeit one that needs suitable adjustment for the relevant mechanical properties of the tissue at the instant of removal [17].

The first aim of this paper is to make a brief survey of what has been established in wear studies in various organisms and to indicate how this information has been used. Secondly, our intention is to suggest how future studies at nanoscale could establish the actual mechanisms of dental hard tissue removal and what questions could be asked. Since most work has been reported on enamel rather than dentine, we restrict our focus here to this tissue.

## 2. Types of Wear Studies

The best method of reviewing the literature seems a classification of method based on scale of observation. *Macrowear* is the surface texture visible on a tooth either with the naked eye or low-power light microscopy. With the use of oblique lighting, shiny-surfaced facets can be distinguished easily from more matte areas on lightly-worn teeth. About 60 years ago, it was established that these patches of wear establish themselves in fairly fixed locations and that the direction of scratches on the shiny facets, evident even if they cannot be individually resolved, could be used to determine how the jaws moved into and out of occlusion [18]. A numbering system for the wear facets on the molars of early mammals was developed [19], which was later expanded to other lineages including the primates [20, 21]. Little has been done since on the evolution of wear in this sense. However, rather than being outdated, macrowear research shows signs of being reinvigorated in several ways. For example, accurate three-dimensional mapping of these facets has been used to reconstruct the jaw movements in prehistoric humans, coupling this to collision detection software to indicate exactly which parts of opposing teeth would have been in contact at any point in time [22]. The results show how the patterns of stress in dental tissues are influenced by the varying direction of the bite force. In addition, macroscopic patterns of postcanine tooth wear have recently been examined with respect to the biological fitness of individual animals [23–29]. Primates that have heavily worn molar crowns consisting only of dentine surrounded by an enamel ridge appear to have very compromised health.

*Microwear* studies were a later development, utilizing the scanning electron microscope (SEM) to observe selected facets of worn teeth at much higher magnifications [30]. Individual features of the worn surface, such as roughly isodiametric pits and elongated scratches, could be distinguished. Microwear has become an extremely important method of determining the diet of fossil vertebrates, particularly when the morphology of the teeth seemed to be of little help in dietary assessment. A glimpse at microwear analysis across vertebrates demonstrates its achievements in a wide variety of contexts. As an example far from modern humans in evolutionary distance, microwear has shown that some of the earliest jawless vertebrates, called conodonts, used their teeth for breaking down large prey items [31]. In our lineage, much of the evidence for what fossil members of our genus were eating comes from dental microwear [32]. However, microwear does not just inform us about food intake. Some fossil primates almost certainly used their procumbent lower front teeth for grooming activities, just as some living primates do. Hair marks between the incisors are clearly evident in both living and fossil groups [33]. Some fossil giraffes, despite their long necks being associated with browsing on the crowns of trees, probably ate grass [34]. Some fossil horses probably did not, despite the fact that they had tall (hypsodont) molar crowns that would seem redundant when eating tree leaves [35]. The discrepancy between the gross anatomy of the dentitions of these ungulates and the microwear evidence of what these ungulates actually seemed to be eating has led to the need for a reexamination of the long-held view that hypsodonty evolved in response to grazing [36]. Despite these successes, the quantification of microwear patterns has proved difficult because SEM provides a two-dimensional view of the surface that is influenced by the direction at which it is viewed. Considerable recent efforts have been made to make microwear analysis more objective by using white-light confocal microscopy to characterize the three-dimensional texture of the worn surface rather than concentrating on individual features such as pits and scratches [37, 38].

*Mesowear* has been an even more recent research development involving light microscopy. It is addressed not to the individual features of worn surfaces, but to their overall roughness and to the curvature of the edges of facets [39, 40]. One of its major aims is to establish whether food or teeth were responsible for the major part of the wear. It would appear that browsers, for example, are significantly different from grazers in this respect [40].

## 3. Causes of Wear

All the above approaches are essentially observational techniques for describing the appearance of worn surfaces. While some experiments have been run to try to determine the causes of particular wear patterns [41–44], the results have been disputed and none have been aimed at demonstrating exactly how tooth material is lost. More broadly, one of the major reasons why research on wear, as a branch of tribology, appears to have been impeded is that the mechanisms that produce it are often unclear. Wear is an accumulation of complex events that can include the combined effects of lubrication, friction, adhesion, and fracture. Practical wear studies often involve a stereotyped kind of apparatus such as a pin on a disk, for which standards are available, but which lack fundamental aims. *In vitro* studies on dental wear have sometimes been very sophisticated. For example, occluding pairs of teeth have been mounted in a universal testing machine, modified to move as does the jaw with a horizontal, not just a vertical, component of motion [45, 46]. This represents a considerable technical achievement in mimicry but does not help necessarily in defining underlying mechanisms.

One way out of such scenarios is to concentrate on the characteristics of individual events. This requires experimentation at the level of nanotechnology. Nanoindentation equipment has only recently started to be used in the equivalent of wear studies. This has provided evidence that enamel tissue is lost far more easily with a sharp contact than a blunt one [47]; that there seems to be a brittle-ductile transition involved in enamel behaviour controlled by force level [48], and that wetting of the tooth surface improves abrasion resistance [49]. However, all these studies have used diamond tips to abrade the enamel rather than the type of mechanical insult to which tooth surfaces are subjected in life. We thus move now to consider what particles could cause damage to dental tissues and in what way such damage might differ from that produced with diamond.

## 4. Potential Sources of Wear

At the level of individual events, mechanical wear can be viewed as a type of indentation involving extensive plasticity. A static indentation may produce a pit, while the addition of some translational motion is liable to lead to a scratch [50]. The major material properties of solids that influence the type of damage that they can inflict on each other are their hardness or yield strength (material resistance to plastic deformation), elastic modulus (material resistance to elastic deformation), and fracture toughness (resistance to crack growth). Hardness is more commonly used than the yield stress in studies, but hardness is strictly a measurement while yield stress is a property. For most geometries of contact, the hardness is about three times the yield stress, but this multiple is not fixed [51]. A basic requirement is that any particle that could endanger dental tissues has to be hard enough to indent the other. A rigid particle in this context is one with a hardness more than 2.5 times that of enamel [51]. Even a particle that is just 1.1 times as hard as enamel would be capable of indenting it, but it would also change shape permanently itself. This situation is called mutual indentation and affects calculations of the force involved in producing a mark of any given size [52].  Table 1 indicates the types of particle that have been suggested as wear agents in the vertebrate literature. All lie in the mutual indentation range with respect to enamel. The most obvious cause of wear is the quartz dust found in many natural environments at various particle sizes [53]. However, phytoliths, the amorphous silica particles found particularly in grasses, are also potential wear agents (Table 1). Less likely candidates as indentation agents are seed shells, against which enamel is effectively rigid.

## 5. Mechanisms of Wear

With the exception of quartz dust, there has been considerable dispute about what kind of particles can wear enamel. For example, phytoliths are thought to be important wear agents by some [54], but not by others [55]. Seed eating has been assumed to be the cause of pitting [37], but this is difficult to understand on the basis of Table 1. In addition there is the issue that tooth tissues wear each other. The only way to resolve these issues would be to initiate wear studies at the appropriate experimental level. This is now feasible via nanoindentation equipment and atomic force microscopy. Such nanowear experiments could focus on individual events, which would erase any doubt as to the nature of the interactions involved.

Prior to making such experiments though, it is reasonable to consider what questions could be asked. These questions could contain the kernels of hypotheses to be tested by nanowear experiments. We offer two examples here with potential answers.

**(1) Why Are Wear Marks So Small?**

It might be thought that this is because** s**maller contacts involve lower forces, so that small marks would be predominant. The problem with this answer though is that a tooth surface usually shows almost no large marks at all. This was why it took an SEM to visualize actual wear features. A more likely response concerns the well-known brittle-ductile transition, which is known to alter the behaviour of small particles [5, 58]. There is some evidence already of such a transition in enamel behaviour [48].

**(2) Why Do Numbers of Pits and Scratches Often not Match?**

**Figure 1:**
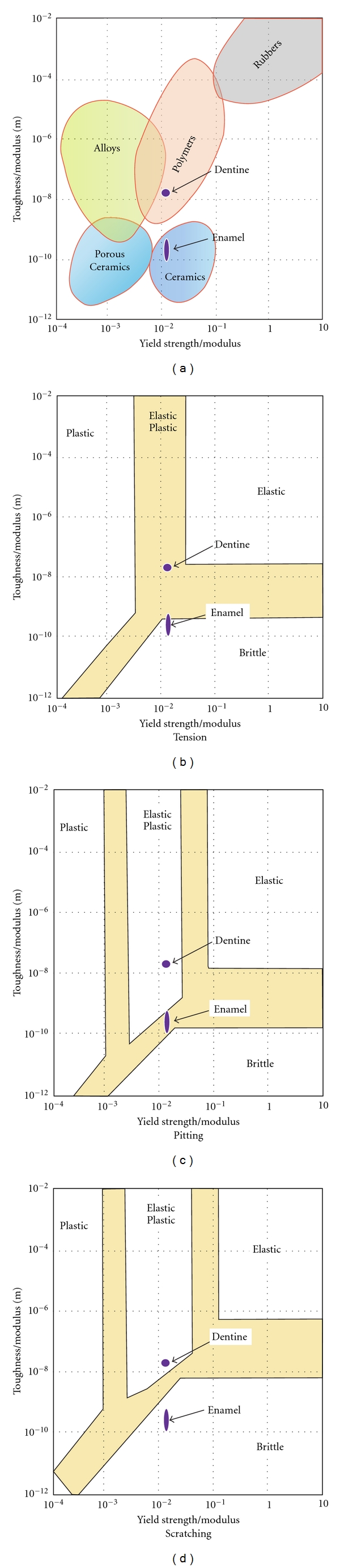
Material property maps (redrawn from [50]). (a) Shows the domains of common types of solids compared to dentine and enamel. Enamel is more like a ceramic in its properties than dentine. Maps (b)–(d) show the effect of different loading regimes on the behaviour of these dental tissues. Under tension (b), enamel is very likely to break in a brittle manner. Under static indentation (c), the highly compressive stresses during pit formation produce a more plastic response, but sliding (d) enhances tension greatly. It can be concluded that enamel is more likely to lose material via a scratch than a pit.

It was noticed from the earliest microwear studies that scratches tend to outnumber pits in most mammals. Jaw movements have been invoked to explain this. For example, in chimpanzees, the third molars, which probably make less of a lateral excursion across each other than more anterior molars, have a higher percentage of pits on their surfaces [59]. However, such considerations do not seem to be sufficient. Rather it would appear that it is likely that scratches form more easily than pits. Some evidence of this comes from considering the mechanics of scratching versus pitting.  Figure 1 is redrawn from Sharp et al. [50] to show the differential propensity of materials to be either scratched (sliding indentation) or pitted (static indentation). The axes of these graphs show the important material properties that influence whether a material will behave either elastically, plastically, or fracture.  Figure 1(a) simply shows how enamel and dentine match up to other materials. Unsurprisingly, enamel is similar to ceramics like glass, while dentine is akin to some polymers.  Figure 1(b) indicates how these materials behave under tension via a subdivision of the map into elastic, plastic, and brittle spaces based on simple equations for tensile behaviour. Again, enamel is brittle, which corresponds with data from tensile tests [1]. However, in Figure 1(c), under the highly compressive stress regime of static indentation, enamel is much less likely to break, showing some plasticity. This is again consistent with data from nanoindentation studies; if the load is small enough, enamel does indeed yield without cracking under static loads. However, when an indenter is slid across a surface to form a scratch, the tensile field behind the indenter is greater and enamel is much more likely to crack (and thus lose tissue) than under a pit. The big difference here is the inclusion of friction in equations for sliding. The frictional coefficient in Figure 1(d) is assumed to be 0.5. Whether such speculations really apply to enamel in “single event,” experiments becomes an important prediction. The time to test such predictions seems to be here.

## Figures and Tables

**Table 1:** Mechanical properties of some materials that might damage enamel.

Particle type	Hardness (GPa)	Elastic modulus (GPa)	Reference
Quartz grit	7–7.75	Not known	[54]
Plant phytoliths	5.8–6 0.5–2.1 GPa	Not known	[54] [55]
Seed shells	0.1–0.4	2–7	[56]
Dental enamel	2–6	50–100	[57]
